# Supplementary material for: Evolution of Food and Nutrition Policy: A Tasmanian Case Study from 1994 to 2023
Source: Nutrients. 2024 Mar 22;16(7):918. doi: 10.3390/nu16070918 (PMC11013732; doi:10.3390/nu16070918)
Supplement: Supplementary file 1 [file nutrients-16-00918-s001.zip › nutrients-2873527-supplementary.pdf]

Table S1. A summary of the four policy and strategy documents that were analysed.

A summary of the title year, government in power, government department, vision and goals, principles, objectives, evidence base, target audience and priority groups, action areas, partners and actors, budget allocation and funding mechanism implementation plans and timelines, evaluation and monitoring mechanisms and policy outcomes and impact indicators for each of the four documents included in the analysis.

Title	**1994 Tasmanian Food And Nutrition Policy [39]**	**2004 Tasmanian Food and Nutrition Policy [40]**	**2012 Food for All Tasmanians—A Food Security Strategy [41]**	**2021 Food Relief to Food Resilience—Tasmanian Food Security Strategy [42]**
**Year**	1994	2004 to 2014	2012	2021 to 2024
**Time frame**	Reviewed every 3 years	Reviewed in 2009	No end point	Action plan delayed 14 months - Jan 2022 to March 2023 to 2025
**Federal Government in power**	Labour - 1983-1996<br>Prime Minister - Hawke-Keating	Liberal - 1996-2007<br>Prime Minister - Howard	Labour Government - 2007-2013<br>Prime Ministers - Rudd-Gillard-Rudd	Liberal – 2013 to 2022<br>Prime Ministers - Abbott-Turnbull-Morrison<br>Labour – 2022 to 2023 at time of publication<br>Prime Minister - Albanese
**Tasmanian Government in power**	Liberal - 1992-1998<br>Premiers - Groom-Rundle	Labour - 1998-2014<br>Premiers - Bacon-Lennon-Bartlett-Giddens	Labour - 1998-2014,<br>Premiers - Bacon-Lennon-Bartlett-Giddens	Liberal – 2014 to 2023 at time of publication<br>Premiers - Hodgeman-Gutwein-Rockliff
**Responsible Tasmanian Government Department**	Department of Community and Health Services (DCHS)	Department of Health and Human Services (DHHS)	Department of Premier and Cabinet (DPAC)	Department of Communities abolished in Feb 2022. Food security function moved to DPAC in Feb 2022
**Vision / Goal / Aim**	All Tasmanians have access to foods that are safe, nutritious, and affordable and that they understand and can choose a healthy diet. It will aim to facilitate and support action through the entire food and nutrition system. It deals with Tasmanian Health issues, economic reliance on primary production, export revenue, specialised food processing, ethnic make-up of community.	Tasmania: a state which produces quality, healthy, safe, and affordable food, while sustaining the natural environment and strengthening the local economy; community a empowered to make food choices that enhance health and wellbeing	All Tasmanians have access to healthy, sustainable, affordable, and appropriate food.	An integrated food relief sector that supports Tasmanians in need to access sufficient, safe, nutritious, quality food, and access services that support long term food resilience. Critical to this goal is the knowledge and expertise of our communities about local need – and fostering the strengths and resources of state-wide and local organisations.
**Goal/Aim**	To make healthy food choices easier and thus reduce the burden of diet-related early death, illness, and disability.<br>To facilitate and support action through the entire food and nutrition system	Endorses the broad goals of Tasmania Together which include:<br>• Goal 1: Ensure that all Tasmanians have the economic capacity to enjoy a reasonable standard of living with regard to food, shelter, transport, justice, education, communication, health and community services.<br>• Goal 5: Develop an approach to health and wellbeing that focuses on	• Achieve better food security outcomes for people and communities most at risk.<br>• Enhance community wellbeing and stimulate economic development through strengthening local food systems.<br>• Embed responses to food security in policy and program development.	An integrated food relief sector that supports Tasmanians in need to access sufficient, safe, nutritious, quality food, and access services that support long-term food resilience.<br>Government and community efforts to increase collaboration, innovation and coordination across Tasmania's food relief sector will be guided by:

		preventing poor health and encouraging healthy lifestyles.<br>• Goal 6: Improve the health and wellbeing of the Tasmanian community through delivery of coordinated service.<br>• Goal 20: Promote our Island advantages including our 'clean green' image, natural resources, location, and people.<br>• Goal 21: Value, protect and conserve our national and cultural heritage.<br>• Goal 23: Ensure there is a balance between environmental protection and economic and social development.<br>• Goal 24: Ensure our natural resources are managed in a sustainable way now and for future generations.		
**Priorities and focus areas**				• Integrated Support – Collaborative leadership and innovation: connection, sector leadership, food literacy and nutrition, local food systems.<br>• Place based – supporting community food resilience solutions: community led co-design, capacity building, strengthening food systems.<br>• Data and information – understanding Tasmanian food relief and food resilience through improved data and information sharing: data gathering, lived experience, understanding systems, governance.
**Focus**	A food and nutrition policy should recognise the need for a food system which is both economically viable and which maintains the quality and integrity of the environment		**I**ncrease access and supply of affordable and nutritious food and community driven approaches to food security for Tasmanians most at risk**.**<br>**The Strategy responds to these basic questions:**<br>• Which Tasmanian groups and places are more likely to be food insecure and why?	The Action Plan acknowledges that every Tasmanian has the right to an adequate standard of living which includes access to food.

			• How can we increase access to wholesome and nutritious food for all Tasmanians but to disadvantaged populations and places?	
**Principles**	As consumers, we are required to choose a healthy diet from an ever-widening array of fresh foods, processed foods, ready-prepared foods, and beverages. The production, processing and marketing of these foods also contributes to the economic well-being of Tasmania and impacts on our environment, our employment, and our future health. It is also recognised that a combination of strategies will be required to protect and develop Tasmania's food supply.	A commitment to:<br>• Environmental sustainability.<br>• Development of the local food industry in recognition of its contribution to the State's economy.<br>• Right of access to a safe and affordable food supply for all Tasmanians.<br>• Consumer participation in the development and implementation of food and nutrition policy and programs.<br>• Prevention and early intervention in relation to diet-related disease and food-borne illness.<br>• A whole-of-population approach to policy implementation, with recognition that some vulnerable groups may require additional focus.<br>• Partnership approaches and collaboration in policy implementation; Innovation.<br>• Evidence-based practice, including monitoring and surveillance.<br>Acknowledgement of:<br>• The influence of global markets on local food production and supply.<br>• The social and cultural factors that influence the eating patterns of individuals and community groups	**Social inclusion, equity, social justice**, including the methods for creating food distribution structures that are fair and responsive to community needs and that support community participation, leadership, and decision-making.<br>**Recognises the need to build:**<br>• **sufficient scale –** i.e. volume capacity to meet need, especially in regional and remote areas.<br>• **scope –** resources and services available to enable at risk groups and places to have the resources and capacity to acquire and use food that is nutritious and balanced.<br>• **sustainability** – the smart use of local assets and ongoing viability of programs and services; and<br>• **connectivity** – mechanisms and opportunities to ensure coordinated effort and interconnections and linkages across the food distribution system.	• **supporting Tasmanians in-need with food relief** that address the determinants of food insecurity.<br>• **Community Driven** - Local communities are best placed to understand local need. They must be informed, connected, and empowered to develop locally based solutions.<br>• **Work together** by collaborating across all levels of Government, the food relief sector and the community sectors is critical to successful local solutions that meet the demand for food relief and drive food resilience.<br>• **Informed and Responsive to Future Need** by Data gathering and information sharing across the food relief sector is required to ensure solutions meet the needs of individual communities and can respond to future demand.
**Evidence basis of the policy actions**	The Tasmanian policy puts the National Food and Nutrition policy (Aug 1992) vision in the context of local food and nutrition initiatives. Providing evidence to link food and nutrition policies arise from importance of food system as a major employer and contributor to the economy, and importance of good nutrition in reducing ill health in the community (Tas Health Goals and	Policy was underpinned by a review of international, national, and state research to underpin best practice.<br>Evidence supporting strategy included Tasmania Together Strategy (2001), Food Standards Australia New Zealand (FSANZ) in the setting of safe food standards and labelling requirements; National Health and Medical Research Council (NHMRC) in the ongoing	Evidence supporting strategy includes social inclusion Strategy 2009, TASCOSS Just Scraping By, Foodscapes Symposium, Anglicare.<br>Take evidence from strong history of food & nutrition policies best practice at Int, National and State level (e.g. WFS 1996, National Food Plan, Local food systems, Victorian Government. Data,	Evidence base from anecdotal evidence, survey responses from emergency food relief geospatial mapping project during COVID-19, data from Tasmanian School Canteens Assoc, Australian Bureau of Statistics, DEX, Tasmanian Government Food Relief Community Reference Group, Tasmanian Government Food Relief Steering Committee, available food relief and food security data from UTAS

	Targets in Tas 1992). Endorses the National Food Authority, NHMRC in developing National Dietary Guidelines, National Farmers Federation, National and international accreditation standards for food manufacturers	development of the Dietary Guidelines for Australians and Nutrient Reference Values. National Public Health Partnership (NPHP) through the Strategic Intergovernmental Nutrition Alliance. The National Food Industry Strategy (NFIS) and the Tasmanian Food Industry Strategy (TFIS),	and outcomes of the Tas Food Security Fund initiatives.	Tasmanian Project prevalence statistics, Foodbank, Interviewing food relief providers, collation of current Tasmanian Govern initiatives impacting food relief including Healthy Tasmania Strategic Plan, Premiers Economic and Social Recovery Action Committee (PESCRAC) which developed a response to COVID 19 and made recommendations in July 2020 and Mar 2021 to enhance food security particularly during emergency situations, evidenced by the development of the Food Relief to Resilience Action Plan informed by the Tasmanian Government's Healthy Tasmania Strategic Plan 2022-26 and the National Food Waste Strategy: Halving Australia's Food Waste by 2030.
**Target audience and priority groups**	Tasmanian population approach	Whole of state approach	Low income, Children, Older people, **Isolated places**	Tasmanians in-need, local communities, communities most reliant on food relief.
**Action areas**	**1) Food and nutrition system –** recognising many different sectors and activities.<br>**2) Monitoring and surveillance** – up to date information about food availability, food intake behaviours, status of health parameters and assessment and incidence of diet-related disease<br>**3) Primary production –** assess quality of foods produced, continuous research, dependence of agrochemicals, control of food borne disease, local production.<br>**4) Environmental aspects** – local producers and manufacturers to establish quality assurance programs, promote local production and consumptions.<br>5) **Manufacturing and processing –** local production to encourage access to healthy food choices, quality assurance to enhance Tas reputation.<br>**6) Quality, safety, technology** – maintain quality assurance	1) **Environment** - To promote practices across the Tas food system that are consistent with environmental sustainability.<br>2) **Food safety –** to ensure the safety of food and drinking water for all Tasmanians.<br>3) **Promote Healthy Eating** for Tasmanians and reduce the impact of diet related diseases.<br>**4) Breastfeeding** – to promote and support breastfeeding in Tas.<br>5) **Food security –** To ensure all Tasmanians have adequate access to nutritious and safe food to meet their nutritional needs. Achieved by increasing awareness, reducing social, cultural, economic barriers, reducing geographical & physical barriers, ensuring special nutritional needs are met.<br>6) **Primary production –** To ensure Tasmania has a primary product sector that is economically vibrant and produces safe and quality food.<br>7) **Distribution, retail, and wholesale** – To Ensure Tasmania has food	1) **Increasing food access and affordability -** support and encourage collaboration between EFR providers, food distributors and food producers to improve state coverage and access for consumers; invest in state-wide coalition model that connect local government, schools, children and families, older people to local, low cost and nutritious food; Establish a regular market basket survey to monitor food prices and availability in disadvantaged communities.<br>2) **Building community food solutions** – Make available tools and resource to support communities to develop skills, and solutions; Invest in state-wide local produce network to support local community school gardens; Resource local government and local community orgs to identify innovative and collaborative solutions.<br>**3) Regional development & support food social enterprises -** Support collaborations that increase opportunities to buy local produce;	1) **Integrated Support –** Connection with successful models including community food hubs, school food programs, hospitality based social enterprise; Sector leadership in food relief provision; Food literacy and nutrition programs; Local food systems – connect state-wide and local food relief providers to local agriculture, hospitality, horticulture etc.<br>2) **Place-Based –** S**upport for community food resilience solutions -** Community led, co-design – support locally driven solutions with relevant gov and community sector activities including school food programs; Work with LGAT to identify councils to lead place-based initiatives; Capacity building with volunteers to identify barriers and ensure sustainable volunteer workforce; Provide access to business and project design support – ie hospitality-based employment and training opportunities through

	programs to enhance Tas reputation.<br>**7) Retailing, wholesaling distribution –** education and training for workers through existing training boards. Nutrition promotion sub-group to promote at point of sale.<br>8) **Labelling –** simplification, inform consumer choice, manufacturers not penalised if unable to include local ingredients.<br>9) **Marketing and promotion** – self-regulation to be investigated, reduce impact on children, encourage implementation of the MAIF agreement, promote wellbeing through healthy eating, promote nutrition at POS.<br>**10) Employment and training** – Ongoing education and training for all workers within the nutrition & food system from e.g. health professionals, UTAS to investigate specialised training and support for students who wish to pursue nutrition careers,<br>**11) Nutrition Education** – Working party to develop a whole community approach. Schools integrate Home Economics and Health Education into curriculum.<br>**12) Catering –** childcare, hospital food service, nursing homes, prisons, take-away food outlets, workplace & school canteens prioritise nutrition.<br>**13) Access and social justice** – strategies to address social justice implications of nutrition, concentrate solely on education but address structural changes to make healthy choices easier choices e.g. services for elderly, parents and other care givers made aware of their	distribution, wholesale and retail systems that are economically viable, safe, and healthy.<br>8) **Food Service –** To ensure the Tas Food service sector is economically viable and provides healthy and safe food.<br>**9) Labelling –** ensure food labelling complies with national requirements and assists consumers to make informed food choices.<br>10) **Media, marketing, and advertising –** Promote healthy food choices, food safety and good nutrition and which promotes locally produced foods.<br>11) **Technology –** Monitor and adopt evidence-based development in food tech e.g. gene technology, food irradiation.<br>**12) Workforce development –** strengthen the capacity, knowledge, and skills of the Tas food and nutrition workforce	Facilitate establishment of food-related social enterprise as part of supporting sustainable local food systems; Promote social procurement by all tiers of government through contractual arrangements.<br>**4) Planning for local food systems by** investing in food sensitive planning - The Tas planning commission and resource management planning commission incorporate food security within framework; Invest in food sensitive urban planning strategies; Strengthen evidence base for food security policy, planning and programs	FR social enterprises; Strengthen food systems to increase community awareness and responsibility for food relief including avenues for donation of in-kind support ie community garden surplus.<br>3) **Data and Information – U**nderstanding Tasmanian food relief and food resilience through improved data and information sharing through data gathering, lived experience, understanding systems, governance - Routine collection of data on distribution of food relief to better understand the needs of community; Undertake regional profiling of food relief and resilience initiatives mapping geographic and social data; Support the collection of info on lived experience of Tasmanians seeking food relief; Undertake an audit of resources that assist food relief orgs to deliver their services, support food resilience and provide referral; Support a community sector alliance to inform Gov decision-making and contribute to community-based solutions and local capacity building. Alliance to liaise with DPAC.

	responsibilities as food producers and role models for children.			
**Partners and actors**	Initiated by Roger Groom Minister for Com & Health Services (CHS),<br>Stage 1 – adhoc working group Gov DoH,<br>Stage 2 – Gov interdepartmental committee inc DCHS, DPAC, DPIF, TDA, OCA, DEA, OW,<br>Stage 3 - Working Group of Gov and Non-Gov<br>1. Kim Boyer, Program Coordinator, Pop Health (DCHS)<br>2. Michele Flint, Executive Officer (DCHS)<br>3. Sonny Azzopardi (CAFTA Tasmania)<br>4. Trevor Beard (Menzies Centre)<br>5. Ted Best (Cadbury-Schweppes)<br>6. Michael Burke (Tasmania, Development and Resources)<br>7. Greg Calvert (DEA)<br>8. Len De Nooyer (Tasmanian Chamber of Retailers)<br>9. Peter France (TFGA)<br>10. Tim Gill, State Nutrition Officer, (DCHS)<br>11. Tim Hohenboken (Summer Kitchen)<br>12. Eric Johnson, Chief Environmental Health Officer (DCHS)<br>13. Malcolm Riley, (Public Health Association)<br>14. Barry Rowe (DPIF)<br>15. Sonia Weidenbach (Office of Consumer Affairs<br>16. David Woodward, (SOM, UTAS)	Initiated by David Llewellyn<br>Stage 1 – Key stakeholder forums attended by diverse range of interested people Aug 2022. Call for submissions in Sep-Oct 2002 came from pre-existing committees or coalitions and other interested groups which formed to develop a submission.<br>Stage 2 – Oct-Nov 2022 Steering committee made up of people interested or expert in food and nutrition. Expert working groups were also established providing advice on specific areas.<br>1. Linda Hornsey (Chair) Secretary, DPAC<br>2. Michael Kent Deputy President, TCCI<br>3. Wesley Hazell Third Rock Agriculture, TFGA<br>4. Tony Demeijer President, Australia United Fresh<br>5. Mark Smith EO, TAPG<br>6. Kevin Baddiley Food Industry Council Tasmania<br>7. Rod Gobbey Director of Agriculture, DPIWE<br>8. Sonia Weidenbach Policy Officer, Consumer Affairs and Fair Trading<br>9. Judy Seal State Nutrition Officer, DHHS<br>10. Madeleine Ball SHS, UTAS<br>11. Julie Williams Coordinator, CNU, DHHS<br>12. Eric Johnson State Food Officer, DHHS<br>13. Graeme Cooksey Principal Education Officer, DOE<br>14. Ros Escott Australian Breastfeeding Association<br>15. Liz Gillam LGAT<br>16. Peter Fehre ED, Retail Traders Association (Tasmania)<br>17. Peta Sugden AGM, Dept of Economic Development	Initiated by the Social Inclusion Commissioner David Adams. The Gov then accepted recommendation to establish a Tas Food Sec Council (TFSC) to oversee development of strategy and fund.<br>TFSC oversaw development of strategy, with social inclusion as core and made recommendations on fund, evaluation, monitoring, advocacy, democratic approach. Members included social inclusion Commissioner, Director of population health, 7 community members appointed by the premier, 2 ex-officio members to represent the state gov.<br>1. David Adams, Social Inclusion Com, Chair<br>2. Roscoe Taylor, D of Public Health, DHHS, Dep Chair<br>3. Janelle Allison, D IRD, UTAS<br>4. Kim Boyer, Rural Health, UTAS<br>5. Sophia Dunn, Ind Consult – Nut & Food<br>6. Jo Flanagan, Anglicare Tasmania<br>7. Michael Gordon, Consult<br>8. Lesley Kirby, D Royal Tas Bot Gardens<br>9. Nel Smit, Comm member, comm gardens	Developed in partnership with food relief sector, community services, and the Tas Government including public consultation, regular meetings of key stakeholders.<br>The following stakeholders provided strategic guidance.<br>1. Anglicare Tasmania<br>2. Eat Well Tasmania<br>3. Families Tasmania<br>4. Foodbank Tasmania<br>5. Hobart City Mission<br>6. Launceston City Mission<br>7. Loaves and Fishes Tasmania<br>8. Local Government Association of Tasmania<br>9. Neighbourhood Houses Tasmania<br>10. Salvation Army<br>11. School Food Matters<br>12. St Vincent de Paul Society of Tasmania<br>13. Tasmanian Council of Social Services<br>14. Volunteering Tasmania<br>15. Commissioner for Children and Young People<br>16. Department for Education, Children and Young People<br>17. Department of Health<br>18. Department of Natural Resources and Environment<br>19. Department of Premier and Cabinet<br>20. Department of State Growth

		18. David Woodward Senior Lecturer, SOM, UTAS<br>19. Tom Ross Food Microbiologist, UTAS<br>20. Lori Rubenstein Policy Development, DHHS<br>21. Roscoe Taylor Director of Public Health, DHHS<br>22. Linley Grant Poverty Coalition<br>23. Wayne John Health Promotion Director, NHF<br>24. Kylie Jackson Policy Officer, DHHS<br>Stage 3 – Community consultation<br>Stage 4 – endorsed by DHHS, DoE, DPAC, DoED, FIC, ABFA, DAA, AUF<br>Stage 5 – Policy Dissemination, promotion and implementation.<br>Stage 6 – policy monitoring		
**Budget allocation**	Not stated	Not stated	$1 million held in trust by the Tasmanian Food Security Council to support eight community driven solutions to improve food security across to areas: 7 projects focussed on innovative responses that have a strategic focus and build on existing capability to address the factors that influence food security (both supply and access to food); secondly project focussed on responses that develop monitoring and surveillance capability to improve the measurements of food security in Tasmania. The eight coalitions involved over 40 organisations and groups from across the state	Strategy 2021-2024 - $3 million in emergency food relief and food security to help the most in-need over 3 years; extend the school lunch program pilot to 30 schools with $1.4 million; increase funding to $60,000 per year to 3 EFR providers for 3 years.<br>Action Plan 2023-2025 details addition $2 million investment in initiatives to support food security. Funding builds on investment of $9 million since 2018 towards the delivery of food relief across Tasmania.<br>- $400k additional funding to school lunch program<br>- $800k allocated to 3 place-based programs funded over 2 years<br>- $100k allocated to nutrition programs<br>- $100k allocated to evaluation
**Funding mechanism implementation plans and timelines**	Not stated	Not stated	The Social Inclusion Strategy for Tasmania and the 2004 Food and Nutrition policy recommended the establishment of a Tasmanian Food Security Council and a Tasmanian Food Security Fund. The TFSC was to advise on the allocation of the fund and to develop a Tasmanian Food Security	A whole-of-government action plan will be developed to implement activity across each of the focus areas identified in the strategy. Developed by Dep of Community in partnership with Food relief Community Reference group and with the strategic guidance of the Food relief Government Steering Committee.

			Strategy. The funding model supported proposals involving a coalition of organisations that range from community, government, local govern and business	Implementation of the Action Plan will be led by the Department of Premier and Cabinet in collaboration with the Food Relief to Food Resilience Steering Committee.
**Evaluation and monitoring mechanisms**	That an appropriate food and nutrition monitoring and surveillance system be established to provide relevant data on the present diet of Tasmanians (including breastfeeding rates) and to assess the incidence of diet-related illness in Tasmania.<br>The establishment of working group to:<br>•• examine the food and nutrition monitoring and surveillance needs of Tasmania;<br>•• further examine potential data sets for a food and nutrition monitoring and surveillance system;<br>•• have input into national discussions on data collection and monitoring and surveillance programs; and<br>•• produce a regular report that provides an overview of the state of the Tasmanian food and nutrition system.	Tasmanian Food and Nutrition policy 2009 progress reports states: Strengthening the capacity for effective monitoring and surveillance to inform action particularly in relation to:<br>-- impacts of food production practices on environmental sustainability<br>-- nutritional status and food intake, including<br>breastfeeding and food security.	The Tasmanian Food Security Council hosted the Foodscapes symposium on 21 November 2011 at the Royal Tasmanian Botanical Gardens. The purpose of the event was to:<br>o hear from and learn from the eight TFSF initiatives and provide an opportunity for coalitions to meet one another, network and share experiences;<br>o learn from best practice interstate examples of food security initiatives; and<br>o consult with the community sector regarding the proposed strategies and priority actions in the Strategy.<br>Foodscapes featured keynote speakers in the areas of community supported agriculture, food sensitive land use planning and local government led community food security projects. Attendees also participated in one of four concurrent workshops aligned with the four strategy areas and priority actions in this document to provide input into its final drafting. A survey was also sent to all Foodscapes participants offering them the opportunity to have further input into the Strategy. The outcomes of the four workshops and the survey have been integrated into this Strategy.	The Progress of this Strategy and its Action Plan will be reported six-monthly to the Minister for Community Services and Development and the Minister for Education and Children and Youth Services and reported annually to Cabinet. The Department of Premier and Cabinet will monitor progress against initiatives in the Action Plan and provide regular updates to the Steering Committee. A final report will be provided to the<br>Minister for Community Services and Development following the conclusion of the Strategy and Action Plan. This final report will inform future work in food relief and food resilience.
**Policy outcomes**	Not stated	Tasmanian Food and Nutrition Policy 2009 progress report states: increased commitment and action across government, community, and private sectors<br>to promote food production practices consistent with environmental sustainability; agreement to establish a Tasmanian Food Security Council with $1 million to be held in trust to support	Outcome of local food systems:<br>raising awareness and understanding in local communities and providing new knowledge of healthy food choices (for example intergenerational projects and cooking classes);<br>o increasing access to affordable healthy food (for example community transport) and local businesses providing	Policy outcomes not available yet.

		initiatives to improve food security; introduction of legislative frameworks for food safety programs; increased awareness and recognition of the importance of healthy eating and the financial burden to the state of chronic lifestyle-related disease; development of the food industry score card to provide reliable information to industry and the government on the value of primary industries from production to consumption; there is a local of local data on the extent of food security in Tasmania;<br>Recommendations to improve and strengthen: policy coordination and awareness, food productivity while addressing climate change and optimising water resource management; food safety in the context of a global food supply; efforts to address the rising prevalence of lifestyle related chronic disease and the ageing population	affordable nutritious meals (using local community produce);<br>o changing food consumption patterns and behaviours (for example children trying fresh produce that they have grown themselves);<br>o increasing local food storage facilities and options (for example fridge purchases in community houses);<br>o increasing sustainable local food supply options (for example mobile fruit and vegetable stalls, farmers' markets and community gardens);<br>o overcoming social barriers and isolation through community connections made with food experiences (for example social eating programs); and<br>o providing jobs, skill development and economic opportunities related to food (for example training for small food producers and opportunities to sell locally).<br>Learning from the TFSF initiatives and through consultations with key stakeholders suggests that these goals can be best achieved by taking simultaneous action on:<br>2.1 Food access and affordability<br>2.2 Community driven solutions to food security<br>2.3 Regional development and food based social enterprise<br>2.4 Planning for sustainable local food systems.	
**Impact indicators**	A number of indicators suggest that the general health of Tasmanians is below that experienced by residents of other Australian states. Heart disease in the north is 26% higher and in the northwest 38% higher than the national average among other chronic diseases including cancer, CVD, dental disease, eating disorders, diabetes, thyroid disease, dietary intake, alcohol, breast feeding rates.	Provided in detail across all of the 12 focus areas	What is clear from all of the TFSF initiatives is that the coalition model of funding is an important point of difference and a challenging but successful approach that has had many powerful outcomes. This model has driven a cross-sectoral integrated approach to community initiatives and has led to new understanding of the role of partnerships to progress food security.	Indicators not available

			This strategy recognises the need to build:<br>o sufficient scale – i.e. volume capacity to meet need, especially in regional and remote areas.<br>o scope – resources and services available to enable at risk groups and places to have the resources and capacity to acquire and use food that is nutritious and balanced.<br>o sustainability – the smart use of local assets and ongoing viability of programs and services; and<br>o connectivity – mechanisms and opportunities to ensure coordinated effort and interconnections and linkages across the food distribution system.<br>The Strategy focuses primarily on the social inclusion aspects of food security including aspects of food supply and food access which impact most directly on vulnerable Tasmanians and where there appear to be local 'solutions'. The Strategy responds to these basic questions:<br>o Which Tasmanian groups and places are more likely to be food insecure and why?<br>o How can we increase access to wholesome and nutritious food for all Tasmanians but in particular to disadvantaged populations and places?	

References

39. DCHS. *Tasmanian Food and Nutrition Policy*; Tasmanian Department of Communities and Health Services: Hobart, TAS, Tasmania, 1994; pp. 1–66.
40. DHHS. Tasmanian Food and Nutrition Policy; Tasmanian Department of Health and Human Services: Hobart, TAS, Tasmania, 2004; pp. 1–58. Available online: https://www.health.tas.gov.au/sites/default/files/2021-12/Food_and_Nutrition_policy_DoHTasmania2004.pdf (accessed on 25 December 2019).
41. TFSC. *Food for All Tasmanians—A Food Security Strategy*; Department of Premier and Cabinet: Hobart, TAS, Australia, 2012.
42. DOC. *Food Relief to Food Resilience—Tasmanian Food Security Strategy 2021–2024*; Department of Communities: Hobart, TAS, Australia, 2021.
